# Menstrual Cycle Symptoms, But Not Oestrogen or Progesterone Concentrations, Are Associated With Sleep in Female Athletes

**DOI:** 10.1002/ejsc.70038

**Published:** 2025-09-16

**Authors:** Madison A. Pearson, Jonathon J. S. Weakley, Alannah K. A. McKay, Suzanna Russell, Josh Leota, Rich D. Johnston, Clare Minahan, Rachel Harris, Louise M. Burke, Shona L. Halson

**Affiliations:** ^1^ School of Behavioural and Health Sciences Australian Catholic University Brisbane Australia; ^2^ Faculty of Health Sciences Sport Performance Recovery Injury and New Technologies (SPRINT) Research Centre Australian Catholic University Brisbane Australia; ^3^ Carnegie Applied Rugby Research Centre School of Sport Leeds Beckett University Leeds UK; ^4^ Mary MacKillop Institute for Health Research Australian Catholic University Melbourne Australia; ^5^ Sport Performance Innovation and Knowledge Excellence (SPIKE) Queensland Academy of Sport Nathan Queensland Australia; ^6^ Performance Services Australian Institute of Sport Bruce Australia; ^7^ School of Psychological Sciences Monash University Melbourne Australia; ^8^ Griffith Sports Science Griffith University Gold Coast Queensland Australia; ^9^ Female Performance and Health Initiative Australian Institute of Sport Canberra Australia; ^10^ Perth Orthopaedic and Sports Medicine Research Institute West Perth Western Australia Australia

**Keywords:** female physiology, hormonal contraception, monitoring, ovarian hormones, sport

## Abstract

This study investigated the associations between ovarian hormones, symptoms, sleep characteristics and nocturnal physiology in female athletes. Twenty‐four National Rugby League Indigenous Women's Academy athletes (naturally cycling: *n* = 11 and mean age: 21 ± 3 years; hormonal contraception: *n* = 13 and mean age: 22 ± 3 years) completed a 5‐week training camp. During the camp, oestradiol and progesterone concentrations were analysed at three timepoints according to naturally cycling and hormonal contraception groups. Symptoms and subjective sleep were measured daily. Athletes were instructed to wear an Oura ring throughout the camp for sleep and nocturnal heart rate (HR) and HR variability (HRV) measures. Statistical analyses included linear mixed models and Pearson's correlations. Neither objective (Oura ring) nor subjective (survey) sleep characteristics were associated with oestradiol or progesterone concentrations. In the naturally cycling group, a higher number of total symptoms were associated with a longer sleep onset latency (*r* = 0.88, 95% CI [0.60, 0.97]) and increased light sleep (*r* = 0.75, 95% CI [0.28, 0.93]). Higher oestradiol concentrations were significantly associated with fewer symptoms (estimate ± SE: −0.007 ± 0.002 symptoms, *p* = 0.003). Luteal days were associated with higher average nocturnal HR and lower HRV than follicular menstrual cycle days (estimate ± SE: 4 ± 0.57 bpm, *p* < 0.001; estimate ± SE: −7 ± 2.13 ms, *p* < 0.001, respectively). Negligible to moderate correlations were observed between sleep and total symptoms experienced by athletes using hormonal contraception. In conclusion, sleep measures were not significantly associated with ovarian hormone concentrations. A higher number of total symptoms were associated with sleep disturbance in naturally cycling athletes. To optimise sleep, female athletes may benefit from monitoring and managing menstrual cycle symptoms.

## Highlights

- Sleep characteristics were not significantly associated with ovarian hormone concentrations in athletes who were naturally cycling or using hormonal contraception.
- Symptoms experienced by naturally cycling athletes across the menstrual cycle had a greater association with poorer objective sleep outcomes than changes in ovarian hormone concentrations.
- Based on this study, monitoring and managing symptoms may be more important for optimising sleep in female athletes than focusing on menstrual cycle‐specific sleep strategies.

## 1 Introduction

Oestrogen and progesterone are ovarian sex steroid hormones that typically fluctuate in a cyclical pattern across the menstrual cycle (Elliott‐Sale et al. 2021). These hormones are regulated by the hypothalamic–pituitary–ovarian axis (Holtzman and Ackerman 2019) and beyond the roles of reproductive function, can influence several physiological processes, including autonomic function and sleep (Pestana et al. 2018; Baker and Lee 2022). A regularly occurring menstrual cycle spans 21–35 days and has been previously described as having four main phases based on ovarian hormone fluctuations (Elliott‐Sale et al. 2021). Alternatively, hormonal contraceptive use suppresses endogenous oestrogen and progesterone concentrations (Hirschberg 2022). Hence, hormonal contraceptive use produces a different hormonal profile according to the type of hormonal contraception used (Elliott‐Sale and Hicks 2018). The central nervous system and the endocrine system are involved in the menstrual cycle, sleep and autonomic regulation; however, little is known about the relationship between sleep and ovarian hormone concentrations. Furthermore, due to an altered hormonal profile when using hormonal contraception (Hirschberg 2022), it is important to consider the potential interactions among hormonal contraception use, hormone concentrations and sleep.

Sleep is associated with multiple physiological processes and supports recovery from physical and cognitive demands (Marshall and Turner 2016). Considering the reports of poor sleep in athletes (Halson 2019), it is important to consider the potential effect of the unique biological ovarian hormone fluctuations on sleep. Polysomnography (PSG) is the gold standard approach for measuring sleep stages (Halson 2019). However, the use of PSG is limited in applied settings due to high equipment costs, the requirement for an appropriately trained sleep technician, a technical and time‐consuming laboratory set‐up and lack of instantaneous feedback (Halson 2019). Consequently, wearable devices have been developed and validated to enable sleep monitoring in the home environment. This can be useful to understand athletes’ sleep characteristics over multiple nights, including sleep across the menstrual cycle.

Female athletes may experience various symptoms throughout the menstrual cycle (Oester et al. 2024; Armour et al. 2020). Common symptoms reported by female athletes include period pain (∼66%) and a range of premenstrual symptoms, such as headaches and mood swings (∼83%) (Armour et al. 2020). A greater number of menstrual cycle symptoms are often reported in the days approaching or during menstruation (Taim et al. 2023), when there is a decline or low concentrations of ovarian hormones (Elliott‐Sale et al. 2021). Due to the prevalence of menstrual cycle symptoms, in addition to the known fluctuations in ovarian concentrations (Elliott‐Sale et al. 2021), sleep quality and quantity may be affected at various timepoints across the menstrual cycle. Healthy women have demonstrated a reduction in subjective sleep quality at the beginning of the menstrual cycle, which typically coincides with more menstrual cycle symptoms (Driver et al. 2008; Baker and Driver 2004). Female athletes have reported decreased subjective sleep quality during the luteal phase compared to the follicular phase (Carmichael et al. 2021). Further, in elite female athletes, menstrual cycle symptoms are associated with both increased sleep duration and greater wake after sleep onset, suggesting a possible link between menstrual cycle symptoms and sleep (Halson et al. 2024). Despite this, the relationship between objective sleep measures and ovarian hormone concentrations at multiple timepoints during the menstrual cycle has received little research attention. Furthermore, the association between sleep and symptoms experienced by highly trained athletes who are naturally cycling and those using hormonal contraception has not been sufficiently investigated. Accordingly, this study aimed to investigate the relationships and between‐group differences for (1) sleep and ovarian hormone concentrations and (2) sleep and symptoms experienced by naturally cycling athletes and those using hormonal contraception and to compare (3) nocturnal physiological responses during follicular and luteal days of the menstrual cycle in naturally cycling athletes.

## 2 Materials and Methods

### 2.1 Participants

Twenty‐four highly trained (tier three, national level) (A. K. McKay et al. 2021) female athletes participated in this study (*n* = 11 naturally cycling and *n* = 13 using hormonal contraception). All athletes were from the National Rugby League (NRL) Indigenous Women's Academy, with further details of the participants reported by A. K. A. McKay et al. (2024). Demographic, sleep and chronotype characteristics are presented in Table 1. Athletes provided informed consent, and the study was approved by the [university name] Human Research Ethics Committee (2021‐285HI) and conducted in accordance with the Declaration of Helsinki.

**TABLE 1:** Demographic, sleep, chronotype and menstrual status or hormonal contraception characteristics.

	Naturally cycling group	Hormonal contraception group	Total cohort
	Mean ± SD (range)	Mean ± SD (range)	Mean ± SD (range)
No. of athletes	11	13	24
Age (years)	21 ± 3 (18–30)	22 ± 3.5 (18–31)	22 ± 3 (18–31)
Height (cm)	163 ± 5 (154–169)	167 ± 3 (160–171)	165 ± 5 (154–171)
Body mass (kg)	71.7 ± 8.4 (59–82)	80.1 ± 13.6 (61–103)	76.3 ± 12.1 (59–103)
Body mass index	27.1 ± 3.4 (21–33)	28.8 ± 4.7 (23–36)	28.1 ± 4.2 (21–36)
Age of the first period (years)	13 ± 2 (9–15)	13 ± 2 (10–16)	13 ± 2 (9–16)
Ovulation day	15 ± 3 (11–20)		
Cycle length (days)	34 ± 6 (26–45)		
PSQI score	7 ± 3 (1–11)	5 ± 3 (0–11)	6 ± 3 (0–11)
Menstrual status or the hormonal contraception type	Evidence of ovulation *n* = 4	Implant (Implanon) *n* = 8	
	Eumenorrheic *n* = 1	Injection (Depo‐Provera) *n* = 1	
	Oligomenorrhea *n* = 3	Oral contraception pill *n* = 4	
	PCOS *n* = 1		
	Anovulatory *n* = 1		
	Luteal phase deficient *n* = 1		

Abbreviations: cm, centimetres; kg, kilogrammes; PCOS, polycystic ovarian syndrome; PSQI, Pittsburgh sleep quality index.

### 2.2 Design

**FIGURE 1:**
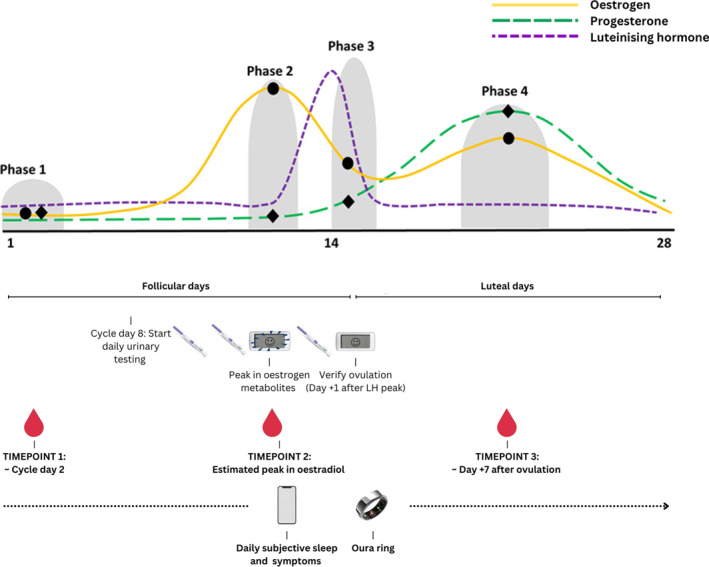
Graphical representation of the research camp testing schedule for naturally cycling athletes. The camp dates for blood collection varied between athletes (depending on the onset of menstruation in naturally cycling athletes). Example of expected ovarian hormone concentration curves adapted from (Elliott‐Sale et al. 2021).

An observational cohort design was used, involving 11 weeks of menstrual cycle characterisation, followed by a 5‐week residential training camp at the Australian Institute of Sport (Canberra, Australia) (A. K. A. McKay et al. 2024). Throughout the camp, athletes completed a daily survey upon waking to report symptoms experienced by naturally cycling athletes and those using hormonal contraception (referred to as ‘symptoms’ from here onward) and perceived sleep quality, using the research electronic data capture system (REDCap) (A. K. A. McKay et al. 2024). During the camp, each athlete was instructed to wear an Oura ring (Generation 3, Oura sleep staging algorithm 1.0, Oura, Finland), and on three occasions, standardised blood sampling procedures for analysis of oestradiol and progesterone were conducted (Elliott‐Sale et al. 2021). Figure 1 illustrates the procedures used in this study across the camp. For further details on methodology and protocols used, refer to A. K. A. McKay et al. (2024).

### 2.3 Procedures

#### 2.3.1 Baseline Questions

Before the camp, athletes completed a series of baseline questions via REDCap. These included a preliminary female athlete questionnaire to assess health background and menstrual status, menstrual cycle history and use of hormonal contraception (A. K. A. McKay et al. 2024) and the Pittsburgh sleep quality index (PSQI) (Buysse et al. 1989). Outcome scores from the PSQI range from 0 to 21, with higher scores indicating poorer sleep. A global score ≥ 5 was used to identify poor sleepers (Buysse et al. 1989).

#### 2.3.2 Daily Monitoring

For 11 weeks leading into the camp, and for the 5‐week camp, athletes completed daily monitoring questions via REDCap (received electronically at 08:00). Athletes rated their sleep quality (on a scale from 1 = insomnia to 5 = very restful), the presence/heaviness of bleeding and the presence of symptoms (selected = yes) experienced during the past 24 h. A total of 16 symptoms options were included: abdominal cramps, acne, appetite changes, bladder incontinence, bloating, breast pain, constipation, diarrhoea, fatigue, headache, lower back pain, mood changes, nausea, night sweats, pelvic pain and sleep changes. Other symptoms could be specified via an open text response and ‘nothing identified’ could be selected if no symptoms were experienced. Further details on the list of symptoms are mentioned elsewhere (A. K. A. McKay et al. 2024).

#### 2.3.3 Sleep and Nocturnal Measures

The Oura ring is a validated (Miller et al. 2022) finger‐worn device containing a triaxial accelerometer and a photoplethysmograph. Athletes were provided with the correct‐sized Oura ring and instructed to wear it throughout the camp and to sync it daily with the Oura ring mobile application (Oura, Oura Health Oy, v4.5). The application was downloaded to each athlete's smartphone before use. Data were exported from Oura Teams platform (Oura on the Web, Oura Health Oy, 2021), and a complete list of variables is available in Supporting Information S1: Supplementary Material A.

#### 2.3.4 Blood Collection

Oestradiol (the predominant form of oestrogen) and progesterone concentrations were assessed from a venous blood draw collected at three timepoints during the camp, dependent on the group. Athletes followed a standardised dietary intake from lunch onward on the day prior to collection and arrived fasted for each collection (Kuikman et al. 2024). In the naturally cycling group, blood collection was scheduled to occur after the onset of bleeding in Phases 1 of the menstrual cycle, Phase 2 when oestradiol peaks and Phase 4 a week following presumed ovulation (phases are outlined in detail by (Elliott‐Sale et al. 2021) and Figure 1). For the hormonal contraception group, samples were separated by seven to 10 days to replicate the pattern of blood collection in the naturally cycling group (A. K. A. McKay et al. 2024). Without instruction from the research team, all athletes who used oral contraception skipped the nonactive pills to avoid a withdrawal bleed during the camp (A. K. A. McKay et al. 2024). Consequently, blood testing occurred on days with assumed exogenous hormone supply for all hormonal contraception users.

In the naturally cycling group, blood collection for Phase 1 occurred approximately one day after bleeding onset when oestradiol and progesterone concentrations are low. Blood collection for Phase 2 aimed to capture a peak in oestradiol, while progesterone concentrations are low. Blood collection for Phase 4 occurred 7 days after presumed ovulation, when progesterone and oestradiol concentrations should be elevated. Dual hormone ovulation kits (Advanced Digital Ovulation Test, Clearblue, Geneva, Switzerland) were used before and during the camp to detect a rise in oestrogen metabolites and surge in luteinising hormone (LH) (A. K. A. McKay et al. 2024). The dual ovulation kits were used from Cycle Day 8 to detect elevated oestrogen metabolites and until a surge in LH was detected (A. K. A. McKay et al. 2024). If an LH surge was not detected, the use of ovulation kits was discontinued on Day 17 of the menstrual cycle (A. K. A. McKay et al. 2024). An arbitrary ‘day 21’ was used for Phase 4 if presumed ovulation was not detected (A. K. A. McKay et al. 2024).

While blood sampling was intended to be in Phase 2, challenges in accurately capturing this timepoint meant that Phase 2 could not be truly confirmed according to the guidelines reported by Elliott‐Sale et al. (2021). As serial blood samples were not collected in the days before Phase 2, the ‘peak’ in oestrogen concentrations could not be confirmed, which is outlined by A. K. A. McKay et al. (2024). Instead of referring to the three specific menstrual cycle phases, three timepoints are used (one, two and three) in order of the menstrual cycle with three different hormonal profiles (timepoints are specific to each group, respectively). In this study, for naturally cycling athletes, the menstrual cycle was also broadly categorised into the follicular and luteal days, based on presumed ovulation (Elliott‐Sale et al. 2021). ‘Follicular days’ refer to any days from the onset of menstruation until the day before presumed ovulation, while ‘luteal days’ refer to any days after presumed ovulation until menstruation. Given the nature of a 5‐week camp, this time frame included days from two menstrual cycles for most naturally cycling athletes (when the luteal phase occurred first). Consequently, the terms follicular and luteal ‘days’ are used, rather than follicular and luteal ‘phases’ to indicate this difference.

#### 2.3.5 Ovarian Hormone Analysis

A trained phlebotomist collected approximately 8.5 mL of blood for each test. Blood serum separator tubes were then left to clot at room temperature for 30 min before being centrifuged at 2200 G for 10 min at 4°C (A. K. A. McKay et al. 2024). The remaining serum was divided into aliquots and stored at −80°C until later batch analysis could be completed. Oestradiol (pg/mL) and progesterone (nmol/L converted to ng/mL) concentrations were measured using the Access Two Immunoassay System (Beckman Coulter, Brea, CA, USA). Retrospective analysis of ovarian hormone concentrations was used to assess the menstrual cycle phase of naturally cycling athletes (Elliott‐Sale et al. 2021; de Jonge et al. 2019); criteria used to assess athletes blood hormone concentrations are summarised in Supporting Information S1: Supplementary Material B.

### 2.4 Statistical Analysis

Statistical analyses were conducted using R Studio (v2023.12.0+369; R Foundation for Statistical Computing, Vienna, Austria). Descriptive statistics, including means, standard deviations (SD) and minimum and maximum ranges were calculated for athlete characteristics, sleep, hormone concentrations, total nights of sleep and total symptoms. Linear mixed models, fitted using restricted estimated maximum likelihood (REML) with 95% confidence intervals (CI), were used to assess ovarian hormone data and the relationships between ovarian hormone concentrations, oestradiol (pg/mL) and progesterone (ng/mL), and objective and subjective sleep measures. Each day when ovarian hormone concentrations were measured, the corresponding prior night of sleep was used in the model, with model estimates, standard error (SE) and the statistical significance provided (significant *p* < 0.05). Linear mixed models were used to examine differences in nocturnal physiological measures (HR [bpm] and HRV [rmssd (ms)]) between follicular and luteal menstrual cycle days. *t*‐tests based on the Satterthwaite's method were used for calculating degrees of freedom and determining statistical significance (*p* < 0.05) using the lmer function in the lmerTest package in R. Fixed effects were included in the group (naturally cycling/hormonal contraception), whereas participant ID was treated as a random intercept term, with Oura ring sleep and physiological measures as the fixed effects. Comparisons using linear mixed models were made between the two groups where suitable. Sleep measures and total symptoms were averaged across the duration of the camp for each athlete, and the Pearson's *r* correlation was provided for each sleep measure in each group (naturally cycling athletes and those using hormonal contraception). The correlations between objective and subjective sleep measures with total symptoms were analysed using Pearson's *r*, and the following descriptive terms were used to support inferences: strong *r* = 0.70–0.89, moderate *r* = 0.40–0.69, weak *r* = 0.10–0.39, and negligible *r* = 0–0.09 (Schober et al. 2018).

## 3 Results

### 3.1 Hormonal Profiles

Background characteristics of all athletes are shown in Table 1. Details on the types of hormonal contraception used are found in Supporting Information S1: Supplementary Material C, including brands and hormonal concentrations.

Results of oestradiol and progesterone concentrations are reported below. In naturally cycling athletes, progesterone concentrations were significantly greater at Timepoint 3 than Timepoint 1 (*p* < 0.001) and 2 (*p* < 0.001). Additionally, oestradiol concentrations were significantly greater in Timepoint 3 than Timepoint 1 (*p* = 0.001). The average hormonal concentrations of oestradiol and progesterone for naturally cycling athletes and those using hormonal contraception are shown in Table 2.

**TABLE 2:** Serum oestradiol and progesterone concentrations across three timepoints in the naturally cycling and hormonal contraception group.

	Naturally cycling group	Hormonal contraception group
	Mean ± SD (range)	Mean ± SD (range)
Timepoint 1		
Oestradiol (pg/mL)	37.9 ± 22.9 (20.3–100.9)	76.1 ± 81.1 (7.8–292.9)
Progesterone (ng/mL)	0.9 ± 1.5 (0.2–5.3)	0.7 ± 0.8 (0.04–2.7)
Timepoint 2		
Oestradiol (pg/mL)	113.5 ± 115.6 (27.9–431.1)	65.4 ± 67.4 (6–230.5)
Progesterone (ng/mL)	0.9 ± 1.4 (0.2–5.2)	0.6 ± 0.6 (0.07–2.4)
Timepoint 3		
Oestradiol (pg/mL)	164.9 ± 112.8 (30.8–422.7)^a^	94.6 ± 178.5 (11.8–682.3)
Progesterone (ng/mL)	9.9 ± 11 (0.2–40)^a^ ^,^ ^b^	0.6 ± 0.5 (0.1–2.1)

*Note:* Timepoints for the naturally cycling group were ordered based on Phases 1, 2, and 4 of the menstrual cycle, whereas timepoints for the hormonal contraception group were separated by 7–10 days in date order of the camp.

^a^ Significantly different to Timepoint 1 in naturally cycling athletes.

^b^ Significantly different to Timepoint 2 in naturally cycling athletes.

### 3.2 Baseline Sleep

Results from the PSQI are summarised in Table 1. Generally, athletes were classified as poor sleepers with 91% (*n* = 10 out of 11) of naturally cycling athletes and 77% (*n* = 10 out of 13) of athletes using hormonal contraception reporting PSQI scores of ≥ 5.

### 3.3 Objective Sleep Measures

A total of 560 nights of sleep were monitored during the camp with the Oura ring (mean ± SD: 23 ± 7 nights/athlete). From all the nights available for sleep monitoring, approximately 77% of nights were monitored with the Oura ring. For the nights corresponding to blood collection days (*n* = 72), approximately 89% of total nights were monitored with the Oura ring (*n* = 64). No significant relationships were found between objective sleep measures and oestradiol and progesterone concentrations (all *p* > 0.05) for the entire cohort, between groups (naturally cycling athletes and those using hormonal contraception), and within naturally cycling athletes across the menstrual cycle (refer to Supporting Information S1: Supplementary Material D). In addition, refer to Supporting Information S1: Supplementary Material E for the mean and standard deviations of Oura ring sleep measures on the night corresponding to each timepoint, in each group.

### 3.4 Subjective Sleep

No significant relationships were found between daily subjective sleep quality and oestradiol and progesterone concentrations (*p* > 0.05) in either group or between groups. Collectively for the total athlete sample, subjective sleep quality improved across the duration of the camp (estimate ± SE: 0.01 ± 0.004 rating, *p* = 0.007).

### 3.5 Daily Symptoms

**FIGURE 2:**
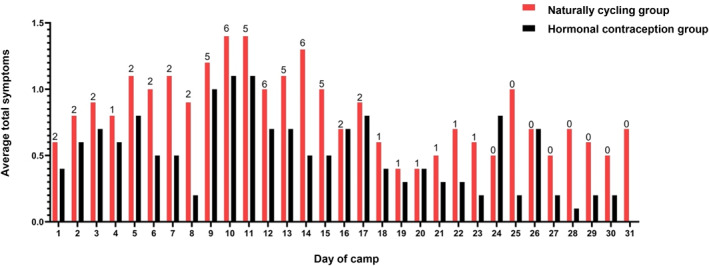
The average number of total symptoms each day across the camp experienced by naturally cycling athletes and those using hormonal contraception. The values above each bar for naturally cycling athletes represent the number of athletes who reported bleeding that day.

A total of 733 daily symptom surveys were completed throughout the camp, which represents a compliance rate of 99.8%. In total, 474 symptoms were reported by the cohort, with 272 symptoms (∼57%) reported by naturally cycling athletes (mean ± SD: 25 ± 38 symptoms) and 202 symptoms (∼43%) reported by athletes using hormonal contraception (mean ± SD: 16 ± 17 symptoms). The most common symptoms reported in the naturally cycling group included fatigue, abdominal cramps, and mood changes, whereas in the hormonal contraception group, the most common symptoms included lower back pain, abdominal cramps and headaches (A. K. A. McKay et al. 2024). The total sum of symptoms reported during the entirety of the camp ranged from zero to 128 symptoms in naturally cycling athletes and ranged from zero to 55 symptoms in athletes using hormonal contraception. The average number of symptoms logged each day in both groups and the number of naturally cycling athletes who experienced bleeding is shown in Figure 2. In addition, higher oestradiol concentrations (pg/mL) were significantly associated with fewer total menstrual cycle symptoms in naturally cycling athletes (estimate ± SE: −0.007 ± 0.002 symptoms, *p* = 0.003).

### 3.6 Symptoms and Sleep Measures

At the between‐athlete level, higher total symptoms were strongly correlated with longer average sleep onset latency (*r* = 0.88, 95% CI [0.60, 0.97]) and longer average light sleep duration (*r* = 0.75, 95% CI [0.28, 0.93]) for naturally cycling athletes. For athletes using hormonal contraception, a higher total number of symptoms were moderately correlated with longer average total sleep (*r* = 0.44, 95% CI [−0.15, 0.80]) and average duration in bed (*r* = 0.47, 95% CI [−0.11, 0.81]). Only in naturally cycling athletes were strong correlations found between sleep and symptoms. All between‐athlete correlations between total symptoms and mean objective sleep measures for both groups are presented in Table 3. Subjective sleep had negligible relationships with total symptoms: total cohort *r* = −0.01, 95% CI [−0.42, 0.39], naturally cycling group *r* = 0.06, 95% CI [−0.56, 0.64] and hormonal contraception group *r* = 0.08, 95% CI [−0.50, 0.60].

**TABLE 3:** Correlations between sleep measures and total symptoms (on average).

Sleep measures	Pearson's correlation coefficient (*r*) (95% confidence limits)	Correlation description
Naturally cycling group		
Sleep efficiency (%)	0.07 (−0.55, 0.64)	Negligible
Sleep onset latency (s)	0.88 (0.60, 0.97)	Strong
Total sleep (s)	0.23 (−0.43, 0.73)	Weak
Duration in bed (s)	0.10 (−0.53, 0.66)	Weak
REM sleep (s)	−0.49 (−0.84, 0.16)	Moderate
Deep sleep (s)	−0.59 (−0.88, 0.02)	Moderate
Light sleep (s)	0.75 (0.28, 0.93)	Strong
Awake (s)	−0.06 (−0.63, 0.56)	Negligible
Midpoint time of sleep (s)	0.41 (−0.26, 0.81)	Moderate
Hormonal contraception group		
Sleep efficiency (%)	0.03 (−0.53, 0.57)	Negligible
Sleep onset latency (s)	−0.09 (−0.61, 0.48)	Negligible
Total sleep (s)	0.44 (−0.15, 0.80)	Moderate
Duration in bed (s)	0.47 (−0.11, 0.81)	Moderate
REM sleep (s)	0.08 (−0.49, 0.60)	Negligible
Deep sleep (s)	−0.03 (−0.57, 0.53)	Negligible
Light sleep (s)	0.30 (−0.13, 0.80)	Weak
Awake (s)	0.09 (−0.49, 0.61)	Negligible
Midpoint time of sleep (s)	0.46 (−0.13, 0.80)	Moderate
Total cohort		
Sleep efficiency (%)	−0.01 (−0.41, 0.40)	Negligible
Sleep onset latency (s)	0.60 (0.26, 0.81)	Moderate
Total sleep (s)	0.20 (−0.22, 0.56)	Weak
Duration in bed (s)	0.20 (−0.23, 0.56)	Weak
REM sleep (s)	−0.23 (−0.58, 0.19)	Weak
Deep sleep (s)	−0.32 (−0.64, 0.10)	Weak
Light sleep (s)	0.64 (0.32, 0.83)	Moderate
Awake (s)	0.04 (−0.37, 0.43)	Negligible
Midpoint time of sleep (s)	0.41 (0.01, 0.70)	Moderate

### 3.7 Nocturnal Physiological Measures

In the 10 naturally cycling athletes where ovulation was confirmed, nocturnal HR (bpm) was higher during luteal nights than follicular nights (estimate ± SE: 3.68 ± 0.57 bpm and *p* < 0.001). Furthermore, nocturnal HRV, measured as rmssd (ms), was significantly lower during luteal nights than follicular nights (estimate ± SE: −7.35 ± 2.13 ms, *p* < 0.001).

## 4 Discussion

The present study aimed to investigate the relationships and between‐group differences for (1) sleep and ovarian hormone concentrations and (2) sleep and symptoms experienced by naturally cycling athletes and those using hormonal contraception and to compare (3) nocturnal physiological responses during follicular and luteal days of the menstrual cycle in naturally cycling athletes. The findings of this study show that there were no significant relationships between ovarian hormone concentrations and measures of objective or subjective sleep in either group. However, in the naturally cycling group, a greater number of symptoms had a strong positive correlation with average sleep onset latency and average light sleep duration. This suggests increased symptoms may be associated with greater difficulty in falling asleep and a lower perceived sleep quality. This relationship was not observed in the hormonal contraception group. Additionally, for the naturally cycling group, the luteal nights of the menstrual cycle were associated with higher HR and lower HRV than follicular nights. Overall, the findings suggest that sleep remains robust to fluctuations in ovarian hormone concentrations in naturally cycling athletes and those using hormonal contraception. However, naturally cycling athletes may be susceptible to sleep disturbances when experiencing an increased number of menstrual cycle symptoms. Therefore, monitoring and managing symptoms may help reduce the potential risk of sleep disturbance.

Oestradiol and progesterone concentrations had no significant relationship with either objectively or subjectively measured sleep across the three timepoints assessed in this study. Increases in progesterone concentrations between presumed ovulation and the mid‐luteal phase in naturally menstruating women have previously been associated with changes in sleep, such as increased Stage 2 sleep measured with PSG (Sharkey et al. 2014). However, in this study, despite the significantly elevated concentrations of progesterone at Timepoint 3 in naturally cycling athletes, these changes in ovarian hormone concentrations were not associated with sleep characteristics. Rather, these findings indicate that sleep may be robust to the menstrual cycle‐induced hormonal fluctuations in our cohort. Across the camp, subjective sleep quality improved for the overall cohort, implying a possible camp effect on sleep, whereby athletes become familiarised to the camp sleeping environment over time.

Relationships were observed between the number of symptoms and select sleep measures in naturally cycling athletes. Specifically, a greater number of total symptoms, on average, in naturally cycling athletes was strongly associated with increased sleep onset latency and increased light sleep duration. This association between symptoms and sleep indicates that naturally cycling athletes who experienced a greater number of symptoms, on average, also had more difficulty in falling asleep and more light sleep. These changes may be due to the subjective discomfort of symptoms, which may potentially disrupt sleep. Furthermore, in naturally cycling athletes, there was a moderate correlation between total symptoms and decreased REM and deep sleep, indicating a lower quality of sleep. Although 83% of our cohort were classified as poor sleepers based on their PSQI scores, this is common among elite athletes (Halson et al. 2022). Nevertheless, this study demonstrates that the symptoms experienced by naturally cycling athletes may be related to sleep characteristics and may also partly explain the higher PSQI scores in this group. This finding agrees with Halson et al. (2024) who found that the total number of symptoms had a greater association with sleep (including increased sleep duration and wake after sleep onset) than the day of the menstrual cycle in naturally menstruating athletes. This suggests that the presence of subjective symptoms may coincide with a greater sleep need or for longer or higher quality sleep. The presence of premenstrual mood disorders has also been shown to increase sleep disturbances (Meers and Nowakowski 2020). Considering that mood changes were a common symptom in the naturally cycling group, this should be considered when tracking symptoms and sleep. Moreover, the naturally cycling group experienced a greater number of symptoms than the hormonal contraception group, which may help explain the stronger correlations found between symptoms and sleep in this group. Whereas more research is needed to understand the effects of hormonal contraception on menstrual cycle symptoms, some evidence indicates that certain types of hormonal contraception may reduce premenstrual‐type symptoms. However, these effects may vary depending on the type of contraception used and severity of symptoms experienced (Coffee et al. 2006; Archer 2006).

The naturally cycling group had a higher average HR and lower HRV associated with luteal days of the menstrual cycle than follicular days. These findings are consistent with both trained (Tier 2) (Ahokas et al. 2023) and untrained healthy women (Alzueta et al. 2022). The inverse relationship between increased average HR and decreased HRV may indicate a reduced parasympathetic response at rest, which may negatively impact recovery during luteal days of the menstrual cycle (Ahokas et al. 2023; Sims et al. 2021). Differences in HR measures have been associated with oestradiol concentrations, although these differences across the menstrual cycle may also be explained by within‐individual differences in ovarian hormone concentrations (Ahokas et al. 2023). Based on this study, naturally cycling athletes should be aware that their average HR and HRV may be adversely impacted during luteal days, particularly when using resting HR and HRV to inform and interpret adaptive responses to physical training.

Although this study is the first to objectively measure both sleep and ovarian hormone concentrations in highly trained naturally cycling female athletes and those using hormonal contraception, it is not without limitations. First, although the sample was representative of a team of female athletes, it is acknowledged that the sample size was small, and caution should be taken if applying findings to a broader population. Second, to remain representative of real‐world female athlete populations, athletes were not excluded if they did not meet criteria for eumenorrhea (refer to Supporting Information S1: Supplementary Material B) (Elliott‐Sale et al. 2021). Furthermore, a variety of hormonal contraception methods were used by athletes within the camp (refer to Supporting Information S1: Supplementary Material C). Future studies with sufficiently large sample sizes could expand on our work by examining whether the type of hormonal contraception moderates associations with sleep. Third, strict comparisons between the four identified menstrual cycle phases could not be determined due to the challenges in accurately capturing all phases as well as the hormonal variability demonstrated in this cohort. Fourth, variable cycle lengths among participants limited the ability to compare objective sleep between groups. Fifth, due to the relational‐based analyses between variables, the causality between variables cannot be concluded from the analyses. Sixth, despite a significant relationship between total symptoms in naturally cycling athletes and oestradiol concentrations, the magnitude of change in oestradiol concentrations that would be considered meaningful is yet to be understood. Seventh, it is acknowledged that high training loads may impact sleep, with likely greater than their average training loads experienced across the camp. Eighth, although a validated device, the Oura ring and the algorithm available at the time of data collection may misclassify sleep stages when compared to laboratory‐based PSG and caution should be applied to the findings relating to multistate sleep (Miller et al. 2022). However, the Oura ring has demonstrated moderate agreement with PSG in multistate sleep classification. This performance was slightly higher than other consumer devices (Miller et al. 2022). The use of PSG requires laboratory‐based, time‐ and resource‐intensive protocols that can interfere with free‐living sleep. In contrast, unobtrusive wearable devices like the Oura ring provide a feasible alternative for continuous sleep tracking in real‐world settings. Finally, within the demands of the camp setting, there were instances of noncompliance with wearing the Oura ring, highlighting the practical challenges of applied research with athletes.

## 5 Conclusion

This is the first study to assess the relationship between sleep and ovarian hormones in highly trained female athletes, across the menstrual cycle and in athletes using hormonal contraception. Whereas no significant relationships emerged between sleep characteristics and hormone concentrations, a greater number of total symptoms, on average, in the naturally cycling group showed a strong correlation with longer sleep onset latency and increased light sleep. Additionally, HR and HRV measures in naturally cycling athletes suggest they experience a reduced nocturnal parasympathetic response on luteal days of the menstrual cycle. The overall findings of this study suggest that sleep in highly trained female athletes is more closely associated with symptoms experienced than oestradiol and progesterone concentrations in naturally cycling athletes. Furthermore, higher concentrations of oestradiol were associated with fewer symptoms in naturally cycling athletes. Therefore, athletes reporting a higher number of total symptoms, particularly those who are naturally cycling, may be more likely to report poorer sleep. Consequently, monitoring and managing symptoms experienced by athletes may be important given the potential influence on sleep.

## Ethics Statement

This study was approved by the Australian Catholic University (ACU) Human Research Ethics Committee (2021‐285HI).

## Consent

Informed consent was obtained from all participants in the study.

## Conflicts of Interest

The authors declare no conflicts of interest.

## Supporting information


Supporting Information S1
