# Supplementary material for: Menstrual Cycle Symptoms, But Not Oestrogen or Progesterone Concentrations, Are Associated With Sleep in Female Athletes
Source: Eur J Sport Sci. 2025 Sep 16;25(10):e70038. doi: 10.1002/ejsc.70038 (PMC12440682; doi:10.1002/ejsc.70038)
Supplement: Supplementary file 1 — Supporting Information S1 [file EJSC-25-e70038-s001.docx]

**SUPPLEMENTARY MATERIAL A**

| **Oura ring measures** | **Description of variables** |
| --- | --- |
| Sleep total (seconds) | Total amount of sleep registered during the sleep period (sleep total = rem + deep + light) |
| Sleep duration (seconds) | Total duration of the sleep period (sleep duration = waketime (sleep bedtime end) to bedtime (sleep bedtime start) |
| Sleep efficiency (%) | Range: 0-100% (100* total sleep/sleep duration) |
| Sleep onset latency (seconds) | Detected latency from bedtime to the beginning of the first five minutes of persistent sleep |
| REM sleep (seconds) | Total amount of REM sleep registered during the sleep period |
| Deep sleep (seconds) | Total amount of deep (non-rapid eye moment stage three) sleep registered during the sleep period |
| Light sleep (seconds) | Total amount of light (non-rapid eye moment stage one or two) sleep registered during the sleep period |
| Awake (seconds) | Total amount of awake time registered during the sleep period |
| Midpoint time of sleep (seconds) | The time in seconds from the start of sleep to the midpoint of sleep based on connected mobile |
| Average heart rate (HR, beats per minute (bpm)) | The average heart rate registered during the sleep period |
| Heart rate variability (HRV, ms) | The average HRV calculated with RMSSD method |

**SUPPLEMENTARY MATERIAL B**

*Menstrual cycle participant classification criteria*

| **Classification type*** | **Criteria** |
| --- | --- |
| Eumenorrheic | - cycle length (based on the last recorded full cycle) >21 days and <35 days, - nine or more consecutive periods per year, - luteinising hormone surge (detected with urinary ovulation kits), - have not used a hormonal contraceptive before recruitment for at least three months, and - serum analyses criteria ^1,21^:   - Phase one: Low levels of oestradiol and progesterone   - Phase two: Oestradiol higher than all other phases and progesterone higher than phase one but <6.36 nmol L^-1^   - Phase four: progesterone concentration >16 nmol L^-1^ |
| Naturally Menstruating | - Regular menstrual flow - Ovulation detected with sensitive kits (Clearblue, Advance Digital Ovulation Test) - Menstrual cycle lengths >21 days and <35 days (cycle length: no. of days between the onset of menstrual flow, day 1 of each cycle) |
| Anovulatory | - No occurrence of LH surge tested with sensitive kits (Clearblue, Advance Digital Ovulation Test) |
| Oligomenorrhea | - Cycle length (based on last recorded full cycle) >35 days |
| Luteal Phase Deficiency | - Phase 4 has an incorrect hormonal profile, refer to the blood analyses section of the study |
| Naturally Menstruating /PCOS | - Meets the criteria outlined for naturally menstruating, although has also indicated polycystic ovary syndrome (PCOS) |
| **Final participant classification occurred after the analysis of serum samples* | |

**SUPPLEMENTARY MATERIAL C**

*Hormonal contraception types*

| Hormonal implant (Implanon) | - Total 68mg Etonogestrel (synthetic progestogen), releases 35-70 μg per day   - Releases small amounts of the hormone and inserted under the skin of the arm   - Long-acting, reversible method (~3 years) - Participants n= 8 |
| --- | --- |
| Hormonal injection (depo provera) | - 150mg/mL of progestin (synthetic progestogen) per injection - Injection is required every three months - Participants n= 1 |
| Oral contraceptive (Evelyn ED) | - 21 active pills   - 30 μg ethinylestradiol (synthetic oestrogen)   - 150 μg levonorgestrel (synthetic progestogen) - 7 non-active ‘placebo’ pills - Participants n=1 |
| Oral contraceptive (Femme-Tab ED 20/100) | - 21 active pills   - 20 μg ethinylestradiol (synthetic oestrogen)   - 100 μg levonorgestrel (synthetic progestogen) - 7 non-active ‘placebo’ pills - Participants n=1 |
| Oral contraception (Linest Ed 30) | - 21 active pills   - 30 micrograms ethinylestradiol (synthetic oestrogen)   - 150 micrograms levonorgestrel (synthetic progestogen) - 7 non-active ‘placebo’ pills - Participants n=1 |
| Oral contraceptive (Yasmin) | - 21 active pills   - 30 μg ethinylestradiol (synthetic oestrogen)   - 3 μg drospirenone (synthetic progestogen) - 7 non-active ‘placebo’ pills - Participants n=1 |

**SUPPLEMENTARY MATERIAL D**

| Relationships between sleep measures and estradiol and progesterone concentrations. For every unit increase in estradiol and progesterone concentrations an estimated change is reported for each variable for: A) the total cohort, B) estimated change in ovarian hormone concentrations between groups, and C) in athletes naturally cycling only. | | | | |
| --- | --- | --- | --- | --- |
|  | Estradiol (pg/mL) |  | Progesterone (ng/mL) |  |
| Oura ring sleep measures | Estimate ± SE | *p* value | Estimate ± SE | *p* value |
| 1. *Total cohort* | | | | |
| Sleep efficiency (%) | 0 ± 0 | 0.95 | 1 ± 2 | 0.69 |
| Sleep onset latency (seconds) | 1 ± 1 | 0.43 | 11 ± 263 | 0.97 |
| Total sleep (seconds) | 5 ± 4 | 0.22 | 149 ± 993 | 0.88 |
| Duration in bed (seconds) | 6 ± 4 | 0.12 | -135 ± 950 | 0.89 |
| REM sleep (seconds) | 5 ± 3 | 0.06 | 453 ± 678 | 0.51 |
| Deep sleep (seconds) | -4 ± 3 | 0.14 | 1063 ± 687 | 0.13 |
| Light sleep (seconds) | 2 ± 4 | 0.61 | -1225 ± 999 | 0.22 |
| Awake (seconds) | 1 ± 2 | 0.61 | -174 ± 434 | 0.69 |
| Midpoint time of sleep (seconds) | 3 ± 2 | 0.24 | -162 ± 494 | 0.74 |
| 1. *Between groups (athletes naturally cycling and using hormonal contraception)* | | | | |
| Sleep efficiency (%) | -3 ± 2 | 0.13 | -2 ± 2 | 0.44 |
| Sleep onset latency (seconds) | 473 ± 342 | 0.17 | 352 ± 322 | 0.28 |
| Total sleep (seconds) | -1058 ± 1337 | 0.43 | -1270 ± 1366 | 0.36 |
| Duration in bed (seconds) | -250 ± 1268 | 0.85 | -1078 ± 1309 | 0.42 |
| REM sleep (seconds) | -1307 ± 1017 | 0.21 | -1177 ± 1057 | 0.27 |
| Deep sleep (seconds) | -842 ± 1400 | 0.55 | 356 ± 1398 | 0.8 |
| Light sleep (seconds) | 998 ± 1471 | 0.5 | -277 ± 1486 | 0.85 |
| Awake (seconds) | 882 ± 553 | 0.12 | 342 ± 548 | 0.54 |
| Midpoint time of sleep (seconds) | -127 ± 642 | 0.84 | -567 ± 657 | 0.4 |
| 1. *Athletes naturally cycling* | | | | |
| Sleep efficiency (%) | 0 ± 0 | 0.16 | -0.5 ± 2 | 0.77 |
| Sleep onset latency (seconds) | -2 ± 2 | 0.40 | -20 ± 264 | 0.94 |
| Total sleep (seconds) | -2 ± 7 | 0.76 | -91 ± 995 | 0.93 |
| Duration in bed (seconds) | -7 ± 6 | 0.25 | 164 ± 952 | 0.86 |
| REM sleep (seconds) | -1 ± 4 | 0.75 | -416 ± 679 | 0.54 |
| Deep sleep (seconds) | 6 ± 4 | 0.13 | -1069 ± 688 | 0.13 |
| Light sleep (seconds) | -5 ± 6 | 0.47 | 1244 ± 1001 | 0.22 |
| Awake (seconds) | -6 ± 3 | 0.08 | 136 ± 435 | 0.76 |
| Midpoint time of sleep (seconds) | -3 ± 3 | 0.42 | 187 ± 495 | 0.71 |
| *pg/mL: picograms per millilitre; ng/mL: nanograms per millilitre; SE: standard error; REM: rapid eye movement sleep; %: percentage of sleep; *p<0.05 indicates a significant relationship* | | | | |

| Mean and standard deviations for oura ring sleep measures on the night corresponding to each timepoint. | | | |
| --- | --- | --- | --- |
| Oura ring sleep measures | Timepoint 1 | Timepoint 2 | Timepoint 3 |
|  | Mean ± SD | Mean ± SD | Mean ± SD |
| *Naturally cycling group* |  |  |  |
| Sleep efficiency (%) | 85 ± 7.53 | 89 ± 5.2 | 89 ± 4.81 |
| Sleep onset latency (min) | 23.8 ± 36.8 | 5.1 ± 2.8 | 15.3 ± 8.8 |
| Total sleep (h.m) | 6.3 ± 1 | 6.6 ± 0.4 | 6.4 ± 1.2 |
| Duration in bed (h.m) | 7.4 ± 1.1 | 7.5 ± 0.4 | 7.3 ± 1.2 |
| REM sleep (h.m) | 1 ± 0.5 | 1 ± 0.3 | 1.1 ± 0.3 |
| Deep sleep (h.m) | 1.6 ± 0.5 | 2 ± 0.4 | 2 ± 0.3 |
| Light sleep (h.m) | 3.2 ± 1.1 | 3.5 ± 0.5 | 3.2 ± 1.2 |
| Awake (h.m) | 1.1 ± 0.4 | 0.5 ± 0.2 | 0.5 ± 0.2 |
| Midpoint time of sleep (s) | 13830 ± 2171 | 13780 ± 909 | 13587 ± 2114 |
| *Hormonal contraception group* |  |  |  |
| Sleep efficiency (%) | 91 ± 4.12 | 89 ± 2.9 | 86 ± 7.1 |
| Sleep onset latency (min) | 10.2 ± 5.1 | 9.1 ± 6.8 | 10.9 ± 6.5 |
| Total sleep (h.m) | 7.1 ± 0.4 | 6.5 ± 0.4 | 6.5 ± 1.2 |
| Duration in bed (h.m) | 8 ± 0.4 | 7.4 ± 0.5 | 7.5 ± 1.1 |
| REM sleep (h.m) | 1.4 ± 0.5 | 1.3 ± 0.5 | 1.3 ± 1 |
| Deep sleep (h.m) | 2.2 ± 1.1 | 2 ± 1.05 | 2.1 ± 1.2 |
| Light sleep (h.m) | 3.1 ± 0.4 | 3.2 ± 0.6 | 3.2 ± 0.5 |
| Awake (h.m) | 0.5 ± 0.2 | 0.5 ± 0.1 | 1 ± 0.3 |
| Average HR (bpm) | 68 ± 8 | 64 ± 7 | 69 ± 9 |
| %: percentage out of 100%; min: minutes; h.m: hours and minutes; s: seconds; bpm: beats per minute. | | | |

**SUPPLEMENTARY MATERIAL E**
